# *Campylobacter jejuni* bile exposure influences outer membrane vesicles protein content and bacterial interaction with epithelial cells

**DOI:** 10.1038/s41598-018-35409-0

**Published:** 2018-11-19

**Authors:** Nayyer Taheri, A. K. M. Firoj Mahmud, Linda Sandblad, Maria Fällman, Sun Nyunt Wai, Anna Fahlgren

**Affiliations:** 1 Department of Molecular Biology, Umeå University, 90187 Umea, Sweden; 2 Umeå Centre for Microbial Research (UCMR), Umeå University, 90187 Umea, Sweden; 3 The Laboratory for Molecular Infection Medicine Sweden (MIMS), Umeå University, 90187 Umea, Sweden

## Abstract

*Campylobacter jejuni* is a prevalent human pathogen and a major cause of bacterial gastroenteritis in the world. In humans, *C*. *jejuni* colonizes the intestinal tract and its tolerance to bile is crucial for bacteria to survive and establish infection. *C*. *jejuni* produces outer membrane vesicles (OMVs) which have been suggested to be involved in virulence. In this study, the proteome composition of *C*. *jejuni* OMVs in response to low concentration of bile was investigated. We showed that exposure of *C*. *jejuni* to low concentrations of bile, similar to the concentration in cecum, induced significant changes in the protein profile of OMVs released during growth without affecting the protein profile of the bacteria. This suggests that bile influences a selective packing of the OMVs after bacterial exposure to low bile. A low concentration of bile was found to increase bacterial adhesion to intestinal epithelial cells, likely by an enhanced hydrophobicity of the cell membrane following exposure to bile. The increased bacterial adhesiveness was not associated with increased invasion, instead bile exposure decreased *C*. *jejuni* invasion. OMVs released from bacteria upon exposure to low bile showed to increase both adhesion and invasion of non-bile-exposed bacteria into intestinal epithelial cells. These findings suggest that *C*. *jejuni* in environments with low concentrations of bile produce OMVs that facilitates colonization of the bacteria, and this could potentially contribute to virulence of *C*. *jejuni* in the gut.

## Introduction

The rapid changes of environmental conditions during passage of microorganisms through the gut forces adaptive modifications to enhance the ability of bacteria to survive. Bile is secreted into the small intestine via the bile duct and plays a major role in the dispersion and absorption of fats from ingested food. Apart from the digestive activity, bile has a confound effect on the microbiome as well as on pathogenic bacteria by having antimicrobial activities, sometimes causing disruption of cellular membranes, protein misfolding and oxidative damage to DNA^1–4^. Instead, enteropathogenic bacteria adapt and respond to the antibacterial activities of bile, *e*.*g*. by modifying outer membrane proteins, using porins, activation of efflux mechanisms, and stress responses. Bile also acts as an environmental signal that affects regulation of bacterial gene expression^2^. This may have significant consequences for the temporal expression of virulence genes by pathogenic bacteria. Notable is also that even closely related bacteria appear to have different responses to bile^2^. The bile concentration in the intestine goes by a steep gradient from being high in the small intestine to very low levels in the large intestine. How different pathogens respond to bile may be influenced by the intestinal localization where they colonize and express their virulence determinants.

The Gram-negative, microaerophilic bacterium *Campylobacter jejuni* is one of the most prevalent food-borne bacterial pathogens and the leading cause of acute human bacterial gastroenteritis throughout the world^5^. *C*. *jejuni* colonizes a wide variety of wild and domestic animals asymptomatically, that also serve as reservoirs for human infection^6,7^. In humans, *C*. *jejuni* colonizes the intestinal tract and can invade the mucosa by crossing the epithelial barriers^8^. The infection manifests clinically in a variety of forms ranging from mild infection to inflammatory diarrhoea with abdominal cramps, fever and vomiting and/or haemorrhagic colitis^5,7,9^. The disease is self-limiting but in rare cases, the infection lead to septicaemia and extra intestinal manifestations like meningitis, cholecystitis, urinary tract infections or septic abortions^7,9,10^. Devastating secondary effects of *C*. *jejuni* infection includes the peripheral neuropathies Miller Fisher and Guillain Barré Syndromes, acute autoimmune neurological disorders that may lead to respiratory muscle compromise and death, arising due to molecular mimicry and antibody cross-reactivity^7^. *C*. *jejuni* infections have also been suggested as one player in the development of Irritable Bowel Syndrome (IBS), Inflammatory Bowel Disease (IBD), and Crohn´s disease^11^.

The pathogenesis of campylobacteriosis is not fully understood, but it is known that the bacteria mainly colonizes the distal ileum and colon where it invades and damages the epithelium and cause an inflammatory diarrhoea^12^. In some cases, translocation to extra-intestinal sites leads to a more disseminated infection. In mice, the highest numbers of bacteria are found in the cecum and proximal colon, while lower numbers and less inflammation are found in the small intestine^13^. Thus, the main colonization and invasion occurs in the lower part of the intestine where the concentration of bile is reduced compared with the proximal small intestine. In cecum, it is estimated that the concentration of bile salts is lower than 1mM^14–16^. During passage in the intestine, *C*. *jejuni* is exposed to bile and thus the ability to resist bile is an advantage in order to survive and establish infection^17^. Accordingly, the bacterium can grow *in vitro* in the presence of 5% ox-bile^18^, although only 35% of bacteria are viable after 24 h culturing in 0.5% bile^19^.

*C*. *jejuni* lacks a type III secretion system, commonly used by many enteropathogenic bacteria for secretion and translocation of virulence proteins that affect host cells by various means. Instead, *C*. *jejuni* uses the flagellar export apparatus to secrete virulence proteins to the surrounding environment or into the host cells^20^. As a complement, outer membrane vesicles (OMVs) appear to serve as an important alternative for the delivery of *C*. *jejuni* effector proteins into host cells^21–23^. OMVs are envelope blebs that consist of outer membrane proteins and lipids, lipopolysaccharides (LPS), as well as periplasmic and cytoplasmic proteins including toxins and virulence factors^24,25^. A number of studies demonstrate important roles for OMVs in bacterial survival, virulence, and pathogenesis, and they can affect host cells even without the requirement of close contact between the bacteria and the host cells^25–29^. Previous proteomic analyses of OMVs from *C*. *jejuni* strain 11168 H identified periplasmic and outer membrane-associated proteins, but also many cytoplasmic proteins known to be important for survival and pathogenesis^22,30^. Interestingly, Elmi and co-workers showed that the OMVs mediated cleavage of E-cadherin and occludin, and thus enhanced bacterial invasion into epithelial cells^23^.

In this study, we analyzed how *C*. *jejuni* 81–176 responds to low bile concentrations in terms of OMV production and protein content, and whether that can possibly affect the virulence of the bacteria. Proteomic profiling showed that the protein content was radically altered in OMVs isolated from bacteria grown in the presence of low bile (0.025%), and this without affecting the overall proteome of the bacteria. Moreover, OMVs from bile-exposed bacteria enhanced adhesion and invasion of bacteria into the epithelial cells.

## Results

### *C*. *jejuni* OMV production is not affected by growth in low concentration of bile

**Figure 1:**
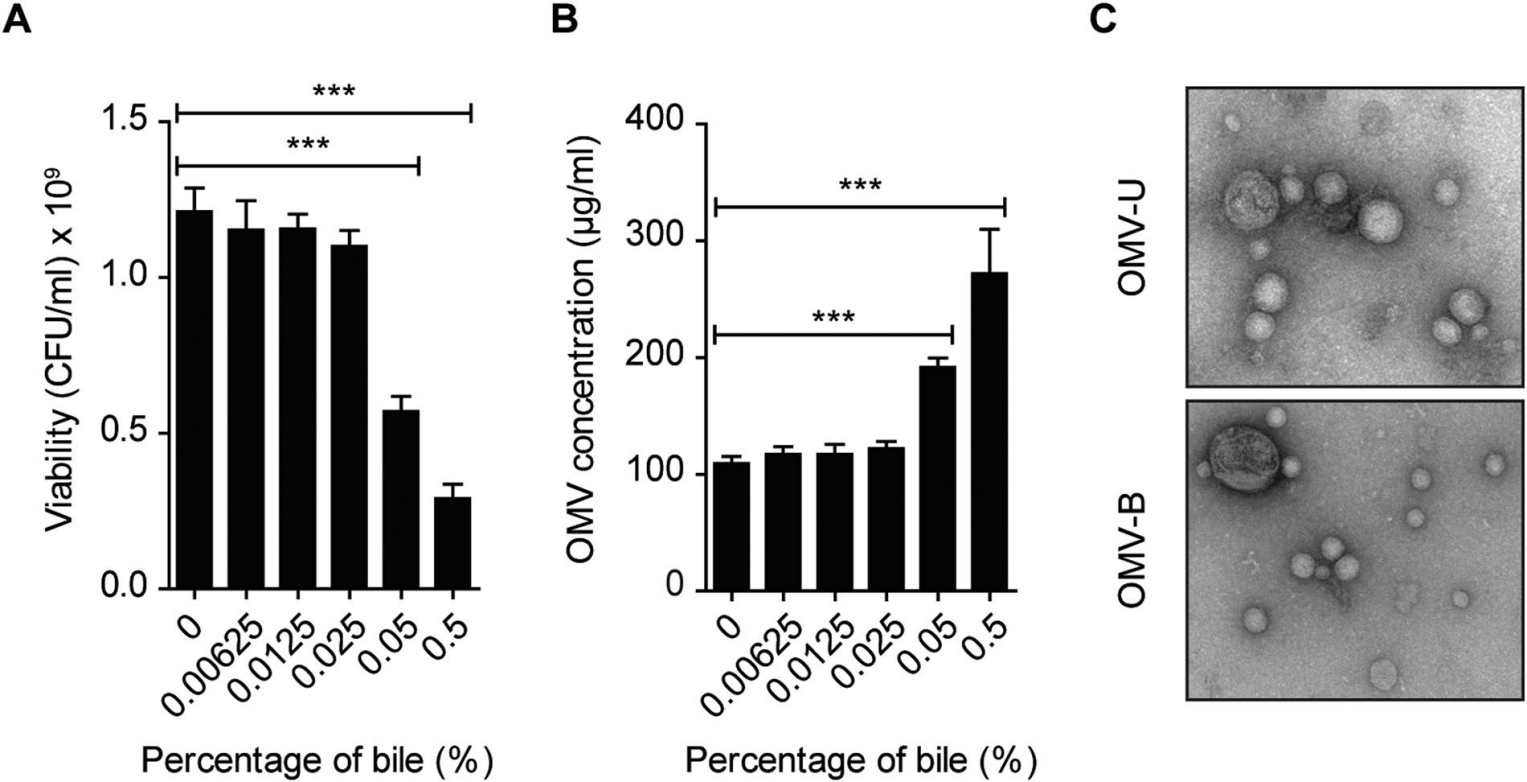
*C*. *jejuni* OMV production is not affected by growth in low concentration of bile. (**A**) *C*. *jejuni* was grown in the presence of ox-bile at different concentrations (0.00625–0.5%) for 20 h at 37 °C and survival was determined by viable count. Medium without bile (0%) was used as control. Data presented as the mean ± SEM for three independent experiments. ***p ≤ 0.001; (one-way ANOVA followed by Bonferroni post-test). (**B**) Concentration of OMVs isolated from *C*. *jejuni* 81–176 grown in different concentration of ox-bile (0–0.5%) for 20 h at 37 °C, quantified by BCA assay. Data presented for three independent experiments. ***p ≤ 0.001; (one-way ANOVA followed by Bonferroni post-test). (**C**) Electron microscopy of OMV-U and OMV-B samples. Pictures are representative of six independent OMV preparations.

The response by *C*. *jejuni* after exposure to bile was analyzed in terms of OMV protein content. Given that OMV production is potentially increased by the detergent traits of bile, we first analyzed bacterial viability and growth and compared with OMV production after exposure to different concentrations of bile for 20 h. For this, *C*. *jejuni* strain 81–176 was grown in the presence of 0.00625 to 0.5% (w/v) ox-bile at 37 °C under microaerobic conditions to resemble the human body temperature and milieu. Ox-bile was selected to reflect the complete bile salt components in the intestine, including sodium salts of cholic, deoxycholic, glycocholic, and taurocholic acids. Although viability was reduced by 53% in 0.05%, and 76% in 0.5% bile, no significant change in viability was seen after 20 h incubation in 0.025% bile (Fig. 1A). Optical density measurments and viability counts examined at 0, 4, 8, 12, 16, 20, and 30 h grown cultures with or without 0.025% bile showed that growth of bile-treated bacteria (WT-B) was only slightly affected by 0.025% bile compared to untreated bacteria (WT-U) at 20 h (Supplementary Fig. S1AB). Next, OMVs were isolated from bacteria grown for 20 h in the different concentrations of bile. There was no significant increase in OMV production by growth in 0.025% bile compared to OMVs from unexposed bacteria, while production of OMVs was significantly increased after exposure to 0.05–0.5% ox-bile (Fig. 1B). Thus, reflecting that reduced viability in response to bile affects OMV production. OMVs isolated from 20 h cultures with 0.025% ox-bile were chosen for further analysis, and here denoted as “low” concentration. The concentration of bile salts progressively decreases along the intestine, estimated to 20 mM in duodenum, 4 mM in ileum, and less than 1 mM in cecum^14–16^. The use of 0.025% ox-bile to study the effects on the OMV proteome in this study is equal to approximately 0.5 mM bile salts, thus in our experimental setting reflecting a cecal milieu.

Nanoparticle-tracking analysis (NTA) was used to measure OMV size and particle number per ml. No difference in particle numbers were detected in OMV samples isolated from bacteria grown in the absence (OMV-U) or presence of low bile (OMV-B) with an average number of 2.7 × 10^9^ particles/ml for OMV-U and 2.6 × 10^9^ particles/ml for OMV-B (Supplementary Fig. S1C). The size distribution of OMV populations ranged between 50–350 nm in diameter in both samples, where there was a slight but not significant increase in the size of OMVs by bile (Supplementary Fig. S1D). The morphology of OMVs was examined by TEM, and revealed similar sizes and morphology of OMV-U and OMV-B (Fig. 1C). Thus, growth of *C*. *jejuni* in low concentrations of bile does not affect production and size distribution of OMVs.

### *C*. *jejuni* alters protein content of OMVs upon exposure to low concentration of bile

**Figure 2:**
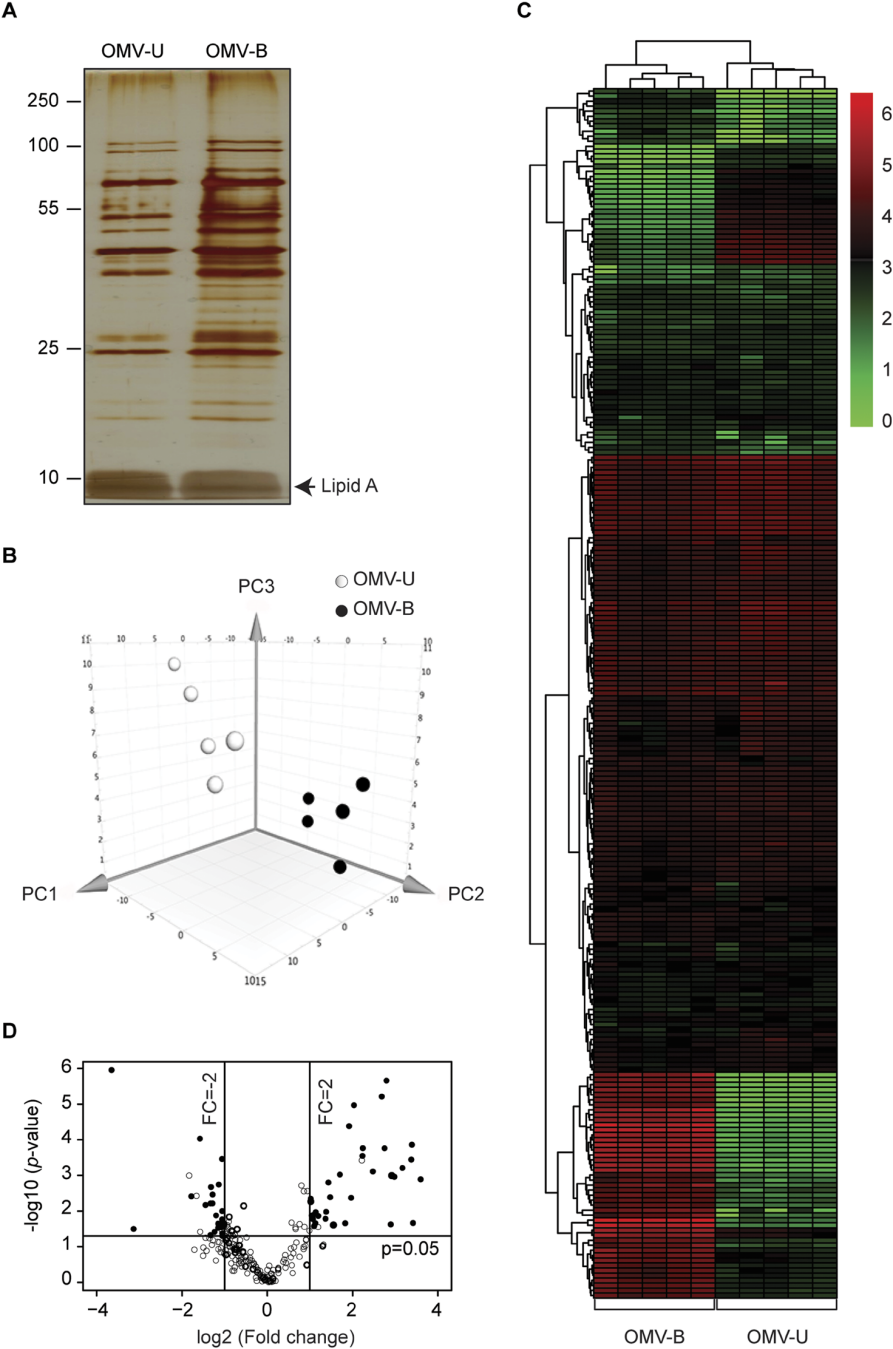
*C*. *jejuni* alters protein content of OMVs upon exposure to low concentration of bile. (**A**) Protein profiles of OMVs isolated from untreated (OMV-U) and bile-treated (OMV-B) *C*. *jejuni* were compared by 12% SDS-PAGE followed by visualization of proteins using silver staining. Lines to the left indicate the molecular masses of the protein standards in kDa. A representative gel is shown. The gel image is adjusted for brightness and contrast by Photoshop, original gel without adjustment and with protein size marker is shown in the Supplementary Data Fig. S4A. (**B**) Principal component analysis showing the relative protein abundance profile of OMV samples in a 3D graph with PC1, PC2 and PC3. The data were mean centered and log transformed. The five biological samples of each type were clustered based on the variance and correlation among them. (**C**) Heat map of a hierarchical cluster analysis of the relative protein abundance profiles of OMV-U and OMV-B samples. The green color represents low and red color represents high expression levels. (**D**) Volcano plot of the complete set of shared proteins detected by proteomic analysis of OMV samples. Each point represents the difference in expression (fold-change) between OMV-U and OMV-B plotted against the level of statistical significance (*p*-value). Solid lines represent differential expression. Empty circles (○) represent not differentially expressed proteins, and black circles (•) represent differentially expressed proteins (*p*-value ≤ 0.05, fold change ≥2, and unique peptides ≥2).

To assess if the protein content of OMVs was affected by low bile, the protein profiles of OMV-U and OMV-B were compared. The same numbers of OMV-U and OMV-B were analyzed by silver staining after SDS-PAGE and showed that the samples differed quite extensively, with OMV-B showing a more complex protein profile (Fig. 2A). To investigate this further, we employed a proteomic analysis of OMV preparations from both conditions (five biological replicates) using LC-ESI-MS/MS. Analysis of the data against the *C*. *jejuni* 81–176 database confirmed that there were significant differences in the protein content in terms of both the number of identified proteins as well as in abundance of proteins between OMV-U and OMV-B. With this analysis, 229 proteins were detected in OMV-U and 237 proteins in OMV-B samples. Among them, 225 vesicular proteins were found in both types of OMV samples, and detected in at least three out of five replicates in each group with high confidence (Supplementary Table S1).

Principal component analysis (PCA) of the five biological replicates for each condition showed a clear separation of the protein profiles of OMV-U and OMV-B and all biological replicates clustered according to their protein abundance (Fig. 2B). Distance within the biological replicates was observed due to certain variability between replicates. A heat map with hierarchical clustering of protein abundance between the two groups clearly showed the correlation of OMV-U and OMV-B samples and individual protein abundance by heat map showed reproducibility of biological replicate within and between groups (Fig. 2C and Supplementary Table S2).

The 225 overlapping proteins detected by LC-ESI-MS/MS were ranked in a volcano plot according to their statistical *p*-value in the form of -log10 (greater numerical value equals a lower *p*-value) versus their fold change (log2). Altered abundance by bile was shown for 56 proteins (*p*-value ≤ 0.05, fold change ≥2.0, and unique peptides ≥2.0). Of these, 33 proteins showed significantly higher, and 23 significantly lower abundance in OMV-B compared with OMV-U (Fig. 2D and Supplementary Table S3).

We next asked whether the observed change in OMVs was a consequence of altered protein content in bacteria. Proteomic analysis using LC-ESI-MS/MS of bacteria grown for 20 h in the absence or presence of low bile (same condition as proteomics of OMVs) identified the same 443 proteins in WT-U and WT-B (Supplementary Table S4). PCA of three biological replicates for each condition showed no obvious distance within the replicates (Supplementary Fig. S2A). Using the same filtering parameters as used for OMVs (*p*-value ≤ 0.05, fold change ≥2.0, and unique peptides ≥2.0); no change in abundance of proteins was observed in bacteria grown in the presence of low bile (Supplementary Fig. S2B). Thus, *C*. *jejuni* 81–176 respond to low concentration of bile by altering the protein composition of OMVs only.

### Bile exposure results in OMVs enriched in cytoplasmic proteins

**Figure 3:**
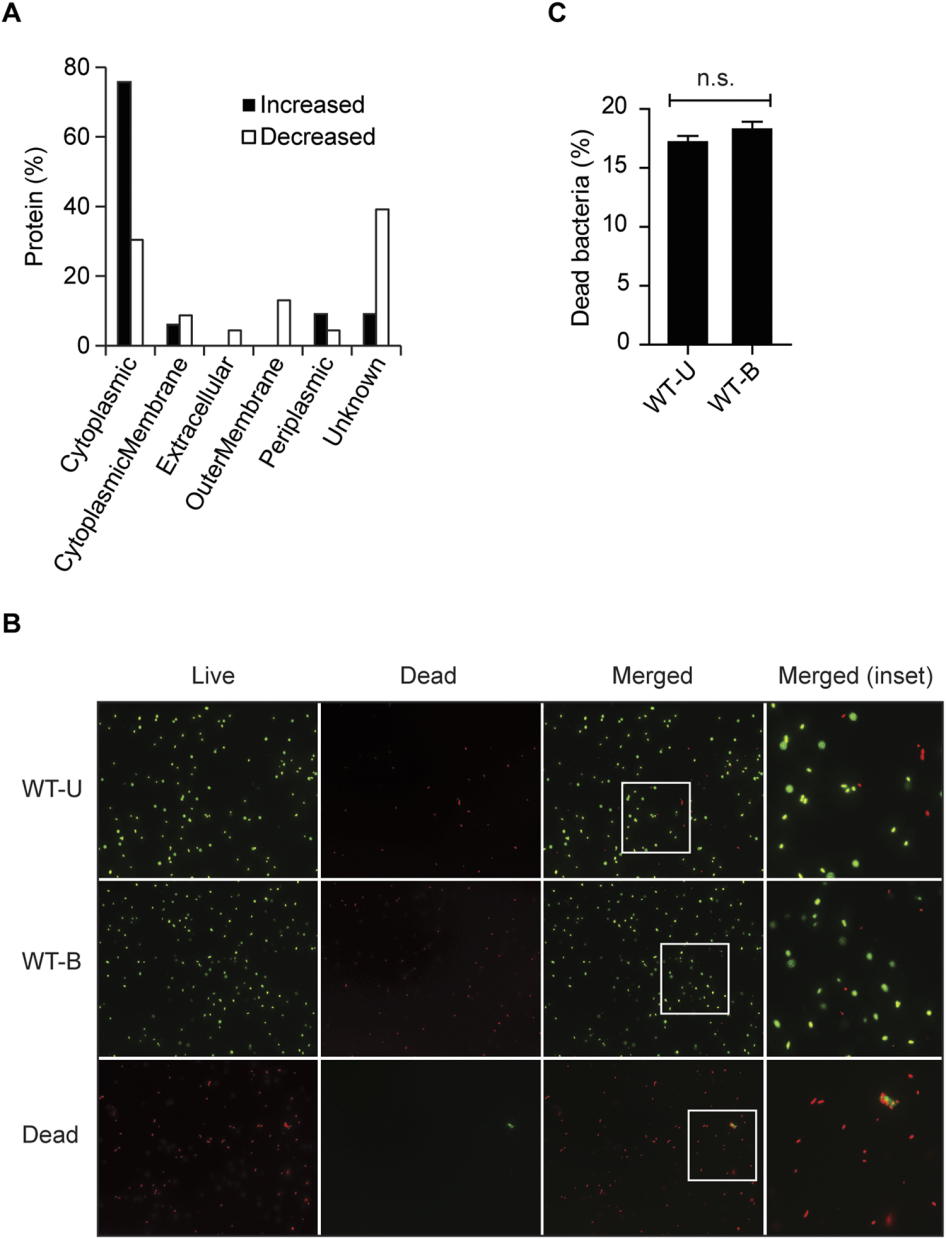
Bile exposure results in OMVs enriched in cytoplasmic proteins. (**A**) Subcellular localization of differentially regulated OMV-associated proteins. The proteins identified through LC-ESI-MS/MS profiling were processed using PSORTb to predict their main location in the cell. Increased; higher abundance, Decreased; lower abundance. (**B**) Fluorescence microscopy analysis of *C*. *jejuni* after live/dead staining. Staining was performed using the LIVE/DEAD® BacLightTM Bacterial Viability Kit L13152 with bacterial samples from *C*. *jejuni* grown at 0% (WT-U) or 0.025% ox-bile (WT-B) for 20 h. Heat-killed *C*. *jejuni* (20 min at 100 °C) was used as dead bacteria (Dead) for comparison. (**C**) Quantification of dead bacteria in *C*. *jejuni* culture grown at 0% (WT-U) or 0.025% ox-bile (WT-B). The numbers of dead bacteria are presented as the percentage of the total counted bacteria (N = 150, WT-U; N = 200, WT-B). Values are presented as the mean ± SEM of three independent experiments n.s.: not significant; (non-parametric Mann-Whitney test).

In an attempt to characterize the alterations of OMV content that were found, the proteins were functionally classified and categorized by the predicted subcellular localization and functional annotation (Supplementary Table S3). The subcellular localization of proteins was analyzed using PSORTb. Among the 33 proteins with increased abundance in OMV-B, 25 (76%) were predicted to be cytoplasmic proteins, three (9%) to be periplasmic proteins, two (6%) to be located in the cytoplasmic membrane, and three (9%) with unknown location (Fig. 3A). For the 23 proteins that decreased in abundance, seven (30%) were predicted as being cytoplasmic proteins, three (13%) outer membrane proteins, two (9%) in the cytoplasmic membrane, one (4%) periplasmic protein, one (4%) extracellular protein, and nine (39%) of the proteins predicted with unknown location (Fig. 3A). This can be compared with the similar analysis for the 225 shared OMV proteins which showed that 49% of proteins were cytoplasmic (Supplementary Fig. S2C). Subcellular localization analysis of the 443 proteins detected in both WT-U and WT-B showed that 58% were predicted to be cytoplasmic proteins (Supplementary Fig. S2D). To reveal if the release of high numbers of cytoplasmic proteins in OMV-B isolations was a result of bacterial cell lysis by growth in bile, we performed live/dead staining on bacteria. No differences in the amount of dead bacteria were seen between WT-U and WT-B samples (Fig. 3B,C). Thus, bacterial growth in low bile results in OMVs enriched in cytoplasmic proteins, which is not a concequence of cell lysis.

**Figure 4:**
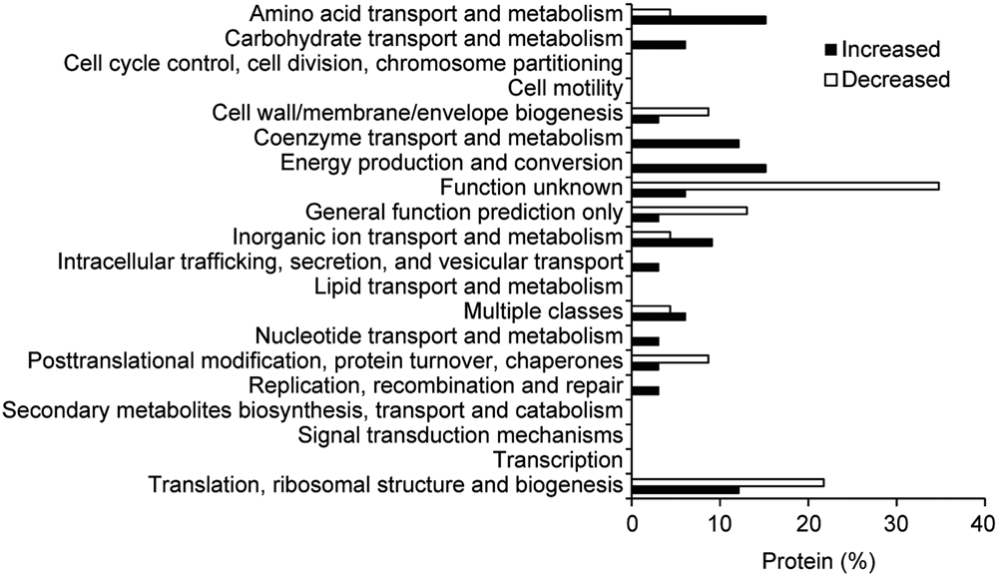
Functional classification of OMV proteins. Classification of differentially regulated OMV-associated proteins using Clusters of Orthologous Groups of proteins (COGs). Increased; higher abundance, Decreased; lower abundance.

To reveal functions of differentially regulated OMV proteins, they were organized according to their Cluster of Orthologous Groups (COG) and grouped into 14 different COG groups (Fig. 4). The majority of the 33 proteins that increased in abundance upon bile exposure were involved in amino acid transport and metabolism (15%), energy production and conversion (15%), translation, ribosomal structure and biogenesis (12%), coenzyme transport and metabolism (12%), and inorganic ion transport and metabolism (9%) (Fig. 4). Of the 23 proteins that decreased in abundance upon bile exposure, the majority of proteins were grouped with uncharacterized function (35%), and translation, ribosomal structure and biogenesis (22%) classes (Fig. 4).

### Bile exposure results in OMVs with reduced known virulence protein abundance

To investigate if proteins with altered abundance could influence virulence, proteins identified by LC-ESI-MS/MS were subjected to *in silico* analysis using MP3, an algorithm developed to predict putative virulence-associated factors. Using this approach, 73 of the 225 proteins detected in both OMV-U and OMV-B were predicted to have pathogenic domain(s) and be potential virulence factors (Supplementary Table S5). Among them, 13 proteins were known virulence factors of *C*. *jejuni*, including the three subunits of CDT toxin (CdtA, CdtB, and CdtC), major antigen protein (Peb1A), CjaC protein, TonB-dependent heme receptor (ChuA), efflux pump subunits (CmeA and CmeC), protein involved in capsule biogenesis (KpsD), fibronectin binding protein (CadF), *C*. *jejuni* surface lipoprotein (JlpA), and flagella proteins (FlaA and FlaB). Furthermore, 10 proteins known to be related to *C*. *jejuni* virulence were not predicted by MP3; Chemotaxis protein (CheV), major antigen proteins (Peb1C, Peb2, Peb3, Peb4), serine proteases (HtrA and CtpA), CjaA protein, and heat shock proteins (GroEL and HtpG). Of these 23 known virulence related proteins, 30% were down-regulated by bile, while 65% were unchanged (Supplementary Table S6). This indicates that the abundance of known virulence proteins of *C*. *jejuni* is lower in OMVs from bacteria exposed to low concentration of bile.

Two proteins with decreased abundance in OMV-B were selected for further analysis. Cell adhesion is the first step in the host-pathogen interactions and adhesion factors are important components of a bacterial infection^31^. CadF is one of the well-studied adhesins in *C*. *jejuni*^32^ and proteomic analysis showed decreased abundance of CadF (2-fold) in OMV-B (Supplementary Table S6). The binding ability of OMV-U and OMV-B to fibronectin were compared and showed no significant differences between OMV-B and OMV-U (Supplementary Fig. S3A). CdtABC is another well-characterized virulence factors of *C*. *jejuni* 81–176 tightly associated with the OMVs^21^ and a genotoxin that cause G2/M phase arrest in epithelial cells^33^. Proteomic analysis showed decreased amounts of CdtABC (3-fold) in OMV-B (Supplementary Table S6) and this was further verified by Western blot analysis (Supplementary Fig. S3B). In order to test if the lower content of Cdt in OMVs after bile exposure affected the state of G2/M arrest, intestinal epithelial cells were stimulated with OMV-U and OMV-B for 24 h and then analyzed by flow cytometry. Although a clear G2/M arrest was visible by the addition of OMV-U, no altered effect was noted for OMV-B (Supplementary Fig. S3C). Possible alteration in the ability of OMV-B to induce cell cytotoxicity was assessed by analyzing cell supernatants for the release of lactate dehydrogenase (LDH). No differences in cytotoxic effect due to exposure to bile were observed after stimulation of intestinal epithelial cells with *C*. *jejuni* or OMVs for 24 h. (Supplementary Fig. S3D).

### OMVs are packed with exclusive proteins upon exposure to low concentration of bile

In addition to the 225 proteins detected in both OMV-U and OMV-B, twelve proteins were detected in OMV-B samples but not in OMV-U samples (Supplementary Table S7). Of these, eight were predicted to be cytoplasmic proteins (GalU, CheA, WaaD, GlmU, SlyD, SerS, GroES, and AcpP), one cytoplasmic membrane protein (CJJ81176_0289), one periplasmic protein (CcoP), one outer membrane protein (CJJ81176_1374), and one with unknown function (CJJ81176_1104). The majority of these proteins were assigned to be involved in cell wall/membrane/envelope biogenesis (Supplementary Table S7). Among the 12 proteins exclusively detected in OMV-B, only Methyl-accepting chemotaxis protein (CJJ81176_0289) was predicted to be associated with virulence (Supplementary Table S7). On contrary, four of the proteins that were detectable in OMV-U, were missing in OMV-B. Three were cytoplasmic proteins (RpmC, RplT, RpsE) involved in translation, ribosomal structure and biogenesis, and one with unknown localization and function (CJJ81176_1184) (Supplementary Table S7). Among these 4 proteins, the conserved domain protein (CJJ81176_1184) was the only one predicted as a virulence protein. Thus, OMVs released by bacteria upon exposure to low concentration of bile appears not only to contain certain proteins with altered abundance, but also to be packed with new proteins.

### OMV-B enhances *C*. *jejuni* adhesion to and invasion into the epithelial cells

**Figure 5:**
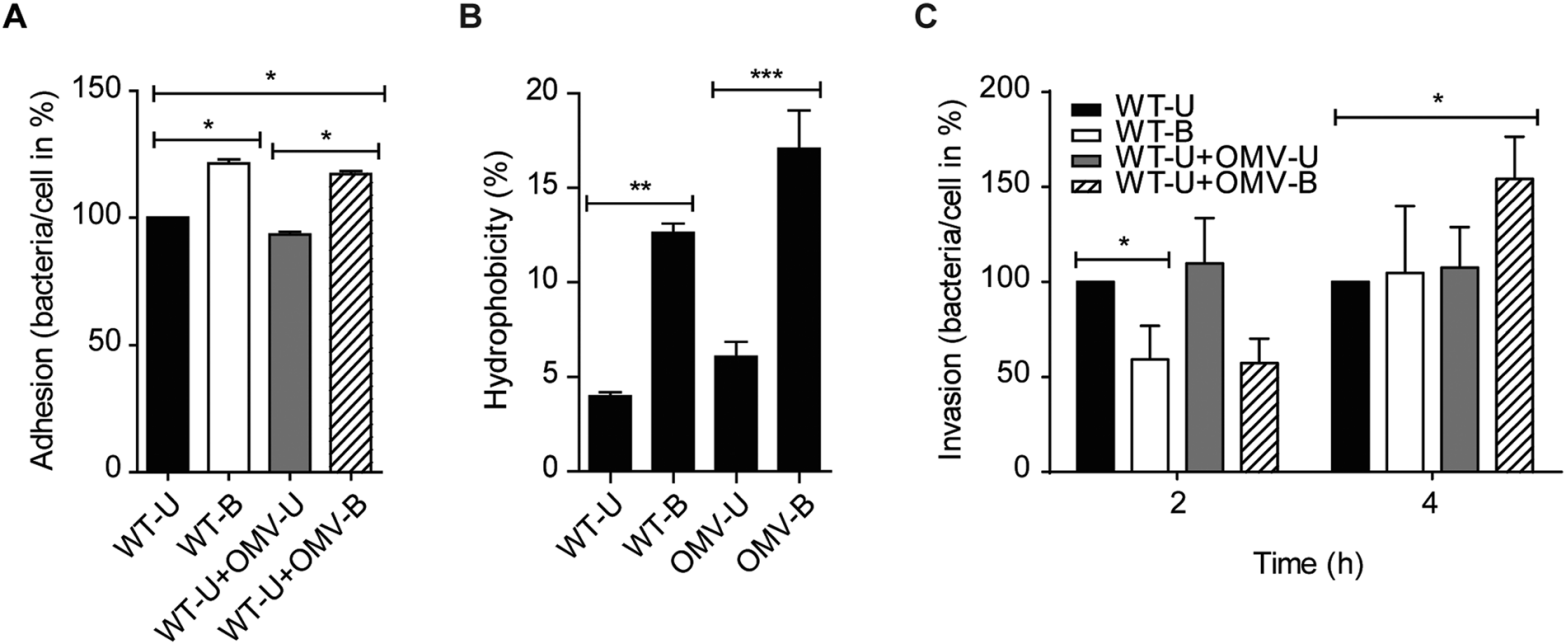
OMV-B enhances *C*. *jejuni* adhesion to and invasion into the epithelial cells. (**A**) OMVs isolated from bile-treated bacteria (OMV-B) enhanced bacterial adhesion to T-84 epithelial cells. Cells were left uninfected or infected with either untreated (WT-U) and bile-treated (WT-B) *C*. *jejuni* at a MOI of 100 or WT-U co-incubated with 10 µg/ml OMV-U or OMV-B for 1 hour at 37 °C. Cells then were lysed and numbers of adhered bacteria calculated and expressed as the number of bacteria/cell in percentage. Data presented as the mean ± SEM for two biological replicates, with each experiment performed in duplicate. *p ≤ 0.05; (non-parametric Mann-Whitney test). (**B**) Cell surface hydrophobicity of bacteria or OMVs was calculated by measuring the affinity of bacteria or OMVs towards xylene. Data presented as the mean ± SEM for three (bacteria) and four (OMV) independent experiments. **p ≤ 0.01, ***p ≤ 0.001; (one-way ANOVA followed by Bonferroni post-test). (**C**) OMV-B enhanced bacterial invasion into T-84 epithelial cells.T-84 cells were left uninfected or infected with either WT-U and WT-B at a MOI of 100 or WT-U co-incubated with 10 µg/ml OMV-U or OMV-B for 1 or 3 h at 37 °C followed by gentamycin (150 μg/ml) treatment for 1 h. T-84 cells then were lysed and numbers of invade bacteria calculated and expressed as the number of bacteria/cell in percentage. Data presented as the mean ± SEM for five independent experiments. *p ≤ 0.05; (non-parametric Mann-Whitney test).

To explore the possibility that OMV-B affects *C*. *jejuni* colonization, we determined if they influenced the adhesion and invasion capacity of the bacteria. For this, adherent T-84 intestinal epithelial cells were infected with WT-U or WT-B for 1 hour, and in parallel WT-U infected cells were co-incubated with OMV-U or OMV-B for the same period of time. It was clear that WT-B adhered to a higher extent to T-84 epithelial cells compared with WT-U (Fig. 5A). Interestingly, co-incubation of WT-U infected cells with OMV-B significantly increased bacterial adherence to the epithelial cells while co-incubation with OMV-U did not (Fig. 5A). This was an interesting observation indicating that OMV-B can affect the adhesive properties of bacteria to intestinal epithelium during infection.

The mechanism behind this is not obvious but given that WT-B showed increased adherence compared to WT-U, the surface properties of bile-exposed membranes could be altered. Thus, we next analyzed if the increase in adhesion was caused by an increased hydrophobicity of the bacterial membrane upon exposure to bile, bacteria were mixed with xylene, and hydrophobicity was calculated. There was a marked increase in affinity towards xylene for WT-B (12.6%) compared to WT-U (3.9%) (Fig. 5B), suggesting that *C*. *jejuni* retrieves an enhanced cell surface hydrophobicity upon exposure to bile. This was also seen for OMV-B in comparison with OMV-U (Fig. 5B). Thus, in addition to altered protein content, exposure to bile results in OMVs with increased membrane hydrophobicity, which likely influences the adhesion of bacteria to epithelial cells.

Given that bile exposure enhanced *C*. *jejuni* adhesive properties, we next examined the role of OMV-B for bacterial invasion into T-84 cells. For this, cells were infected with WT-U and WT-B, or WT-U co-incubated with OMV-U or OMV-B for 2 or 4 h. The invasion capacity was measured by viable counts of intracellular bacteria. After 2 h of infection, invasion of WT-B was significantly lower compared to WT-U, but reached to the same invasion capacity after 4 h infection (Fig. 5C). While the addition of OMV-B had no significant effect on bacterial invasion at 2 h, there was a significant increase of invasion by WT-U at 4 h (Fig. 5C). Thus, *C*. *jejuni* modifies the protein content of OMVs upon exposure to bile, which in turn aids the adhesion and invasion ability of bacteria to the intestinal epithelium.

## Discussion

Release of OMVs by bacteria occurs over the course of normal growth, and different stressors can increase this production. The question rises if the OMVs produced under stress also have an impact on the virulence of bacteria. Here we show that low concentration of bile, which does not affect bacterial viability or OMV production, indeed modulates the OMV protein content, and this influences bacterial adhesion. The low bile concentration used is approximately equal to what is found in ileum and cecum and it is therefore likely that the increased adhesiveness caused by bile in this environment contribute to *C*. *jejuni* virulence.

Although many proteins showed different abundance in the OMV content released by bacteria exposed to low bile, no changes in protein content were detected in the bacterial samples using the same filtering parameters. The bile-exposed OMVs contained 45 proteins with increased abundance of which 12 were exclusive, and not detected in OMVs from untreated bacteria. Moreover, 23 proteins showed decreased abundance in bile-exposed OMVs and 4 proteins were exclusively detected in OMVs from untreated bacteria. On the contrary when the same analysis was performed on whole bacteria untreated or bile-treated, no difference in protein expression was detected.

According to analysis of subcellular localization of OMV proteins, most of the proteins that increased in abundance were cytoplasmic proteins (76% of proteins with increased expression and 67% of the exclusively detected proteins). While it is believed that cytoplasmic proteins should not be present in OMVs because of their biogenesis^34^, several other recent proteomic studies confirm their presence in OMVs from different bacteria, albeit they purified OMVs using a density gradient^22,30,35–37^. In bacteria, more than half of the detected proteins were cytoplasmic, but we could exclude that the high number of cytoplasmic proteins in OMV-B were the result of bacterial cell lysis. Recents studies have shown that OMVs released from Gram-negative bacteria could contain sub-populations of vesicles with both outer and inner membranes^38,39^. These double-membrane OMVs would therfore be able to carry cytoplasmic component including cytoplasmic proteins, DNA and RNA. We suggest that some cytoplasmic proteins may in fact be sorted into the bacterial outer membrane vesicles. Although the mechanism for packing OMVs is still unclear, the increase in cytoplasmic proteins in OMVs upon exposure to bile suggests that bile exposure influence packing of the vesicles. This might involve the different outer and inner membrane properties as well as periplasmic differences of bile-exposed bacteria.

Further, compared to OMVs from unexposed bacteria, bile-exposed OMVs were loaded with lower amounts of proteins predicted as virulence-associated proteins. Since the overall capacity of OMVs to load proteins does not appear to be changed, it may be that bacteria deliberately decrease the amount of virulence related proteins to be associated with OMVs. Thus, we suggest that bile influence selective packaging of cellular components into the OMVs of *C*. *jejuni*. Selective packaging has been suggested in a number of studies of OMVs^40–44^. For example, all three CDT subunits were mainly found in OMVs and not in the bacteria, but compared to OMVs from untreated bacteria there were lower amounts of these subunits in bile-treated OMVs. Bile did however not affect CDT abundance in the bacteria. None of the *Campylobacter* invasion antigens (Cia) were detected in OMVs in our setting, and neither was CiaB detected in OMVs after growth in taurocholate^45^. This is in line with that the flagellum is known to be the secretion apparatus for Cia proteins^20^ and CiaB protein was detected in bacteria only but with no alteration in the protein level by low bile at 20 h. Increased transcription and translation of *ciaB* gene in bacteria was previously shown with high concentration of bile^46,47^.

The protein with highest increase in abundance in bile-exposed OMVs was Cpp47 (hypothetical protein), encoded by the pTet plasmid. This is a 206 aa protein with unknown function and with no obvious domains. There are homologs in many *C*. *jejuni* subspecies and *Campylobacter coli*, but not in other bacteria. Among proteins detected exclusively in OMVs from bile-exposed *C*. *jejuni* are the chemotaxis protein CheA, and two proteins involved in LOS biosynthesis, GalU (UTP-glucose-1-phosphate uridylyltransferase) and GlmU (UDP-N-acetylglucosamine pyrophosphorylase). CheA may play an important role for colonization in mice^48^. GalU is important for resistance to antimicrobial peptides in *C*. *jejuni*^49^, but also in *E*. *coli* for modulation of the TNF-a response in macrophages^50^, in spreading of *Shigella sonnei* from the gut to peripheral organs *in vivo* and resistance to complement-mediated lysis^51^, and in *V*. *cholerae* colonization and resistance to antimicrobial peptides, bile, and complement killing^52^. The consequence of having these proteins selectively packed in OMVs upon bile exposure is not obvious. Further, the CmeABC multidrug efflux pump is important for bile resistance in *C*. *jejuni*^17,53^. CmeA and CmeC were found in OMVs but only CmeA was up-regulated in OMV-B. However in bacteria the efflux pump is functional only if the three subunit is present^54^.

Our data are to some extent different from those recently reported by Elmi and co-workers^45^ studying the effects of taurocholate on *C*. *jejuni* OMVs. First, in our proteomic analysis the OMV-B proteome was compared with OMV-U within the same analysis and where the OMVs were isolated in the same experiments, allowing a direct comparison of sample types, and thereby a higher possibility to detect very low levels of protein. In their study, they did not do this type of direct comparison, only analyzing protein content in OMVs from taurocholate-exposed bacteria. Second, while we were using a concentration of our bile constituent not stressing the bacteria to increase OMV production, the concentration used for taurocholate markedly increased the OMV release. Hence, we have compared the protein profiles of the same number of OMVs and thus specifically detect how bile-exposure affects OMV content and not the situation where bacteria are under stress resulting in increased OMV production. Third, the protein content of OMVs might also differ between different *C*. *jejuni* strains due to genome diversity. The *C*. *jejuni* strain 81–176 used here have 87 unique ORFs compared with *C*. *jejuni* 11168^55^, and also the pTet and pVir plasmids, which are not present in 11168^55–58^. Elmi and co-workers suggested higher abundance of three serine proteases in response to taurocholate^45^. Of these, only HtrA and CtpA (corresponding to Cj0511 in strain 11168) were detected but not altered by bile in OMV-B and WT-B.

Interestingly, the low concentration of bile was found to increase bacterial adhesion to intestinal epithelial cells, an effect also reported for *Bacteroides* and *Bifidobacter*^59,60^. This effect likely has implications for the infection situation where the bile concentrations at the site of colonization are in a similar range. One reason for the increased adhesion is likely that *C*. *jejuni* get an enhanced hydrophobicity of the cell membrane following exposure to bile. It has been reported that bile increase the membrane hydrophobicity in *Bifidobacter* and *Lactobacillus*^61,62^, and higher membrane hydrophobicity has been shown to lead to higher adhesion for *Lactobacillus* strains^63^. Increased hydrophobicity by bile exposure was also seen for *C*. *jejuni* OMVs. The increased hydrophobicity likely contributes to a more pronounced adhesion to the epithelium by both OMVs and bacteria. However, the increase in adhesiveness caused by bile exposure was not associated with increased invasion. The invasiveness was even lower than that seen for the less adhesive non bile-treated bacteria, suggesting down regulation of the invasion machinery in bile-treated bacteria. This has also been seen for *Salmonella enterica* typhimurium that senses bile to repress genes involved in invasion when growing in the intestinal lumen^64,65^. It was noteworthy that OMVs from bile-exposed bacteria could enhance adhesion of bacteria not exposed to bile, reaching similar adhesive capacity as bile-exposed bacteria. It is likely that this is caused by binding of the OMVs to the bacteria facilitating interaction with the epithelial cells. However, in contrast to that seen for bile-exposed bacteria, the enhanced adhesion of untreated bacteria in presence of OMV-B was associated with increased invasion of epithelial cells. This discrepancy in invasion capacity between cell-bound bile-treated and untreated bacteria strongly supports our hypothesis that bile reduces the bacterial invasion potential. Further, the observed adhesion promoting property of OMVs from bile-exposed bacteria might have implications on bacterial infectivity by also promoting adhesion of less adhesive bacteria, and thereby enhance colonization and possible also invasion. This hypothesis can be supported by a previous study showing that *Proteus*, *Acinetobacter*, and *Pseudomonas* could be isolated from mice MLN, liver, and spleen along with *C*. *jejuni*^66^.

Taken together, our study highlights environmental influences on bacterial traits during infection. Here we show that environmental concentrations of bile affect *C*. *jejuni* adhesive characteristics and also the OMV content without influencing the amount of OMVs released, and also that the released OMVs have capacity to enhance adhesion of bacteria not adapted to the environment.

## Materials and Methods

### Bacterial strain and growth conditions

*Campylobacter jejuni* strain 81–176 was cultured on Mueller Hinton Agar (MHA) plates supplemented with vancomycin (10 µg/ml), trimethoprim (5 µg/ml), and polymyxin B (2.5 IU/ml) for 16 h at 37 °C under microaerobic conditions using chemical Gas Pack generator (CGP) (BD GasPak™ EZ gas generating container systems and GasPak EZ Campy Container Sachets 260680)^67^. This system produces a microaerobic atmosphere within 2 h with approximately 6–16% O_2_ and 2–10% CO_2_ within 24 h according to manufacturer. To assay survival of bacteria under bile stress, overnight cultured bacteria were harvested in 1 ml MH broth (MHB) and inoculated in 100 ml MHB supplemented with different concentrations (0, 0.00625, 0.0125, 0.025, 0.05, and 0.5% w/v) of ox-bile (Sigma-Aldrich, St. Louis, MO, USA), and incubated at 37 °C under microaerobic conditions for 20 h (mid-exponential phase). Samples were monitored by optical density (OD600) at different time points and plated on MHA.

### Cell line and culture conditions

Human colon carcinoma cell line T-84 and human ileocecal carcinoma cell line HCT-8 were grown in Dulbecco’s Modified Eagles/F-12 nutrient mixture supplemented with 10% heat-inactivated FCS, L-glutamine (2 mM), and penicillin-streptomycin (100 µg/ml) at 37 °C in 5% CO_2_. Human colon adenocarcinoma cell line LS174T was cultured in DMEM + GlutaMAX supplemented with 10% heat-inactivated FCS, HEPES (15 mM) and penicillin-streptomycin (100 µg/ml) at 37 °C in 5% CO_2_.

### Isolation of outer membrane vesicles

OMVs were isolated from culture supernatants as described previously^21^ with some modifications. Briefly, overnight culture of *C*. *jejuni* were diluted 1:100 in MHB with or without ox-bile and transferred into cell culture flasks with slant layer of MHA (biphasic media) and incubated under microaerobic conditions for 20 h at 37 °C. Bacterial cells were removed from culture media by centrifugation at 5000 × *g* for 30 min. Supernatants were filtered through a 0.22 μm membrane filter to remove cell debries and ultra-centrifuged at 100,000 × *g* for 2 h to pellet down the vesicles. All isolation steps were carried out at 4 °C, and the resulting OMV pellet was suspended in 20 mM Tris-HCl (pH 8.0) and used immediately. OMV samples were plated out on MHA plates and incubated under both microaerobic and aerobic conditions for 48 h to confirm the absence of viable bacteria.

### Measurement of number of vesicle and protein concentration of OMVs

A NanoSight NS300 instrument (Malvern Ltd, Worchestershire, UK) was used for measurement of OMV particle concentration and size as described^68^. Briefly, samples were diluted 1:50 in 20 mM Tris-HCl pH 8.0 buffer and loaded into the sample chamber. Videos were recorded for 60 s and size of individual OMVs and total number of OMV particles were analyzed by Nanoparticle Tracking Analysis software (NanoSight Ltd., UK). All measurements were performed at room temperature. Bicinchoninic Acid (BCA) Assay kit (Thermo Scientific Pierce, Rockford, IL, USA) was used to measure OMV concentration.

### SDS-PAGE and immunoblot analysis

5 µg/well OMV samples were separated by sodium dodecyl sulfate-12% polyacrylamide gel electrophoresis (SDS-PAGE, median size, 130 × 95 × 1 mm, 10 ml) at 80–180 V, 500 mA using Running TG-SDS 10% liquid concentrated Ultarpure buffer (containing 0.025 M Tris, 0.192 M Glycine and 0.1% SDS, VWR). This was followed by staining to detect proteins using Silver stain kit (Thermo Scientific Pierce, Rockford, IL, USA), according to the manufacturer’s protocol or transferring onto PVDF membranes for Western blotting. Nonspecific binding was blocked by incubation of the membranes in milk powder (5%) in Tris-buffered saline pH 8 containing 0.1% Tween-20 (TBST) for 1 hour at room temperature (RT). Membranes were incubated with rabbit polyclonal anti-Cdt-A antibody (1:5,000)^69^ in TBST overnight at 4 °C followed by washing in TBST and incubation with Horseradish Peroxidase-conjugated donkey-anti-rabbit IgG (1:5,000; GE Healthcare, Buckinghamshire, UK) for 1 hour at RT. Amersham ECL kit (GE Healthcare, Buckinghamshire, UK) was used for detection.

### Transmission electron microscopy (TEM)

OMVs were adsorbed to glow discharged carbon coated copper grids and negatively stained with a solution of uranyl acetate (1.5%). The samples were examined on a Phillips CM120 Biotwin transmission electron microscope operating at 120 kV at 17500× magnification. Micrographs were recorded with a Olympus SIS Cantega G2 2k CCD camera using iTEM software.

### Protein extraction and digestion

40 µg of total proteins in OMV samples were extracted largely following published procedures^70^, apart that the digestion was performed on a 10 kDa spin-filter, as that allowed the final concentration of sodium deoxcycholate (SDC) during digestion to be reduced without increasing the volume. The resulting peptides were cleaned-up using a C18 STAGE-tip^71^. 10 µg of bacterial proteins were extracted and trypsin-digested closely following published procedures^70^, followed by a post digestion clean-up on an Oasis^®^ HLB µElution plate (Waters, Massachusetts, USA). The eluate was diluted 1:50 to lower the organic content to about 1% before MS analysis. For each condition, five biological replicates were used for OMVs and three biological replicates for bacteria.

### Liquid chromatography-electrospray ionization-tandem mass spectrometry (LC-ESI-MS/MS)

The OMV protein samples were loaded on an HSS T3 C18 analytical column (75 μm i.d. × 200 mm, 1.8 μm particles; Waters, Milford, MA), and separated using a linear 108 min gradient of 5–41% solvent B (3:1 Acetonitrile/2-propanol) balanced with 0.1% aqueous formic acid (solvent A) at a flow rate of 295 nl/min. The eluate was passed to a nano-ESI equipped Synapt^TM^ G2-Si HDMS mass spectrometer (Waters, Milford, MA) operating in resolution mode. All data were collected using ion-mobility MS^e^ with a scan-time of 0.5 sec and mass-corrected using Glu-fibrinopeptide B and Leucine Enkephalin as reference peptides. Approximately 700 ng of each bacterial protein sample were loaded on a BEH C18 analytical column (75 μm i.d. × 250 mm, 1.7 μm particles; Waters, Massachusetts, USA), and separated using a concave 180 min gradient of 1–40% solvent B (0.1% formic acid in acetonitrile) with 0.1% aqueous formic acid as solvent A, using a flow rate of 368 nl/min. The eluate was passed to a nano-ESI equipped SynaptTM G2-Si HDMS mass spectrometer (Waters, Massachusetts, USA) operating in resolution mode. MS^e^ data were collected using a scan-time of 0.4 sec, mass-corrected using Glu-fibrinopeptide B and Leucine Enkephalin as reference peptides.

### Proteomic data processing and analysis

For OMVs, MS/MS spectra were searched using Progenesis QI software (Nonlinear Dynamics, Newcastle, UK), peptides where identified using the built in MS^e^ search option, employing a false discovery rate (FDR) less than 1% with a mass error cut-off of 10ppm. Two missed cleavages where allowed, carbamidomethylated Cysteines as fixed modification, oxidized Methionine, deamination of Asparagine and Glutamine as variable modifications. Protein abundances were calculated using the Hi-N method^72^ in the software, with N = 3. The data for bacterial proteomic analysis were processed with Protein Lynx Global Server v.3.0.3 (Waters, Massachusetts, USA) and the resulting spectra were searched against the *C*. *jejuni* 81–176 subsection from Uniprot (2017_06). The database search settings were: enzyme-specific cleavage with 1 miss-cleavage allowed; carbamidomethylated cysteines as fixed modification; oxidized methionine, N-terminal acetylation and deamidated asparagine and glutamine as variable modifications. A minimum of three fragments where required with a peptide and fragment tolerance 10 and 25 ppm, respectively; with a false positive rate of 1%. A single data dependent run using a pooled sample containing equal parts from the 6 biological samples were run as verification for the MS^e^ identifications, and databank searched using an in-house Mascot server (Matrix Science Ltd, UK). Peptides with high quality identification i.e. a PLGS score ≥6 and identified by Mascot, where imported into Skyline v3.6 (MacLean, Tomazela *et al*. 2010) filtered and mapped to the raw data. The final data matrix was evaluated using MSStats package in R (Choi, Chang *et al*. 2014). Protein abundances were calculated using top 3 matching peptides.

### Bioinformatic analysis of proteomic data

To visualize the relative protein abundance profiles of OMV biological samples, principle component analysis (PCA) and hierarchical clustering followed by heat map were done by SIMCA 14.1 software (Umetrics AB, Umeå, Sweden) and R 3.3.2 software (https://www.r-project.org/), respectively. The data were mean centered and log transformed. The biological samples were clustered based on the variance and correlation among them. Proteins present in at least three of five replicates of OMV samples, were considered as abundant, and a *p*-value ≤ 0.05 by ANOVA was considered significant. To visualize the relative protein abundance profiles of bacterial biological samples, principle component analysis (PCA) was done by SIMCA 14.1 software (Umetrics AB, Umeå, Sweden). The data were mean centered and log transformed. The biological samples were clustered based on the variance and correlation among them. Proteins present in at least two of three replicates of bacterial samples, were considered as abundant, and a *p*-value ≤ 0.05 was considered significant. The subcellular locations of the identified proteins were analyzed using PSORTb version 3.0.2 (http://www.psort.org/psortb)^73^. The functional annotation of the identified proteins were categorized according to the COG annotation terms by using COGsoft^74^. Prediction of virulence-associated proteins was performed using MP3 software (http://metagenomics.iiserb.ac.in/mp3/)^75^. Theoretical pI (isoelectric point) and Mw (molecular weight) for each protein was calculated using ExPASy - Compute pI/Mw tool (https://web.expasy.org/).

### Hydrophobicity Assay

Hydrophobicity assay was performed as described by Iwata *et al*.^76^ with some modifications. *C*. *jejuni* was grown in MHB at 37 °C for 20 h, pelleted by centrifugation at 5000 ×* g* for 15 min, and washed twice with phosphate buffer saline (PBS, pH 7.0). OD600 of the bacteria was measured and adjusted to ~1.0 (initial value = in). One ml of bacterial suspension was added to 1 ml of xylene, vortexed vigorously for 30 seconds and kept at RT for 30 min for phase separation. The OD600 of the bacterial suspension (aqueous phase = aq) was measured and the percentage of cell surface hydrophobicity was calculated as [(OD in – OD aq)/OD in] × 100. Hydrophobicity of OMVs was determined by measurement of protein concentration (using BCA) before and after adding to xylene and the percentage of hydrophobicity was calculated as [(BCA in – BCA aq)/BCA in] × 100.

### Adhesion and invasion assay

T-84 cells were seeded over night at a density of 1 × 10^5^ cells/well in 12-well plates. Next day, the cells were washed twice with PBS and infected with *C*. *jejuni* at a MOI of 100, with or without co-incubation with OMVs (10 µg/ml) in antibiotic free cell media. For adhesion assay, cells were incubated for 1 hour at 37 °C followed by washing three times gently with PBS to remove unattached bacteria. For invasion assay, cells were incubated for 1 or 3 h at 37 °C followed by gentamycin (150 µg/ml) treatment for 1 hour. Afterwards, cells were gently washed three times with PBS to remove gentamycin. Cells were lysed in Triton-X (0.2%) and bacterial counts were determined on MHA plates after serial dilution.

### Cell cycle analysis

HCT-8 cells were seeded over night at a density of 3 × 10^5^ cells/well in 6-well plates. Next day, the cells were washed twice with PBS and infected with *C*. *jejuni* at a MOI of 100 or stimulated OMVs (10 µg/ml) in antibiotic free cell media at 37 °C in 5% CO_2_. Cells treated with nocodazole (1 µM) served as a positive control. After 1 hour of infection, gentamycin (100 µg/ml) was added for 1 hour and replaced by 10 µg/ml for 24 h. Cells were then detached, washed in PBS supplemented with 5% heat-inactivated FCS, and treated with Vindelöf-PI (50 µg/ml propidium iodide in 50 mM Tris, 100 mM NaCl, 0.1% NP-40, and 20 µg/ml RNase) on ice for 30 min. The PI intensity was measured by flow cytometry (Guava, Merck Millipore, Darmstadt, Germany), using InCyte software.

### Cytotoxicity Assay

LS174T cells were seeded over night at a density of 5 × 10^4^ cells/well in 24-well plates. Next day, the cells were washed twice with PBS and infected with *C*. *jejuni* at a MOI of 100 or OMVs (10 µg/ml) in antibiotic free cell media for 24 h at 37 °C. Cell supernatants were analyzed for the release of lactate dehydrogenase (LDH) using the CytoTox 96 nonradioactive cytotoxicity assay kit (Promega Corporation, Madison, WI, USA), according to the manufacturer’s protocol. Untreated cells represented the 0% cytotoxicity (negative control) and total lysis of cells following treatment with Triton X-100 (1%) represented the 100% cytotoxicity (positive control). The percentage of cytotoxicity was calculated as 100 × (LDH released/total LDH).

### Fibronectin binding assay

Fibronectin binding assay was performed as described previously^77^ with some modifications. Briefly, a sterile 96-well plate was coated with fibronectin (10 μg/ml) or BSA (20 μg/ml) overnight at 4 °C. Next day, wells were washed with PBS and OMVs (50 µg/ml) were added to the wells and incubated at RT for for 4 h. The wells were subsequently washed with PBS and then incubated with rabbit anti-OMP50 antibody (1:500)^78^ for 3 h, followed by washing and incubating with Alexa Fluor 488-conjugated donkey-anti-rabbit IgG (1:500; Invitrogen, Paisley, UK) as secondary antibody at RT for 1 h. After washing the wells, 100 µl PBS was added to the wells and fluorescence was measured with an excitation wavelength of 488 nm and an emission wavelength of 519 nm in Tecan Infinite M200 Microplate reader (Tecan Group Ltd., Männedorf, Switzerland).

### Live/dead staining and fluorescence microscopy analyses

Live/dead bacteria cell staining was performed using the LIVE/DEAD^®^ BacLight^TM^ Bacterial Viability Kit L13152 (Molecular Probes, Inc, Eugene, OR, USA) according to the manufacturer’s instructions. Briefly, *C*. *jejuni* grown in 0% or 0.025% bile for 20 h was harvested, and washed twice in 0.85% NaCl by centrifugation. Bacterial suspension of 10^7^ bacteria/ml were subjected to staining. Heat-killed bacteria (100 °C for 30 min) was used as a population of only dead cells for comparison. The staining was analyzed in a Nikon fluorescence microscope with bandpass filters Fluorescein and Texas Red for live and dead bacteria, respectively.

### Statistics

Statistical analyses were performed using Prism statistical software (V6.05; Graph Pad Software, San Diego, CA). One-way ANOVA followed by Bonferroni post-test, non-parametric Mann-Whitney test, or Student *t*-test was performed to test differences among different groups. Differences with a value of *p* ≤ 0.05 were considered statistically significant. The densitometric analysis of Western blot was performed using ImageJ 1.48 v (NIH).

## Electronic supplementary material

Figure S1-S4

Dataset S1-S7

## Data Availability

The datasets generated and/or analyzed in the current study are included in the article (and its Supplementary Information Files) or available from the corresponding author on reasonable request.
