# Supplementary material for: Campylobacter jejuni bile exposure influences outer membrane vesicles protein content and bacterial interaction with epithelial cells
Source: Sci Rep. 2018 Nov 19;8:16996. doi: 10.1038/s41598-018-35409-0 (PMC6242867; doi:10.1038/s41598-018-35409-0)
Supplement: Supplementary file 1 — Figure S1-S4 [file 41598_2018_35409_MOESM1_ESM.pdf]

Supplementary Material

Campylobacter jejuni Bile Exposure Influences Outer Membrane Vesicles Protein Content and Bacterial Interaction with Epithelial Cells

Nayyer Taheri[1,2], AKM Firoj Mahmud[1,2,3], Linda Sandblad[1,2,3], Maria Fällman[1,2,3], Sun Nyunt Wai[1,2,3], and Anna Fahlgren[1,2,*]

[1]Department of Molecular Biology, Umeå University, Umea, Sweden

[2]Umeå Centre for Microbial Research (UCMR), Umeå University, Umea, Sweden

[3]The Laboratory for Molecular Infection Medicine Sweden (MIMS), Umeå University, Umea, Sweden

[*] **Correspondence:** Dr. Anna Fahlgren, anna.fahlgren@umu.se

Figure S1

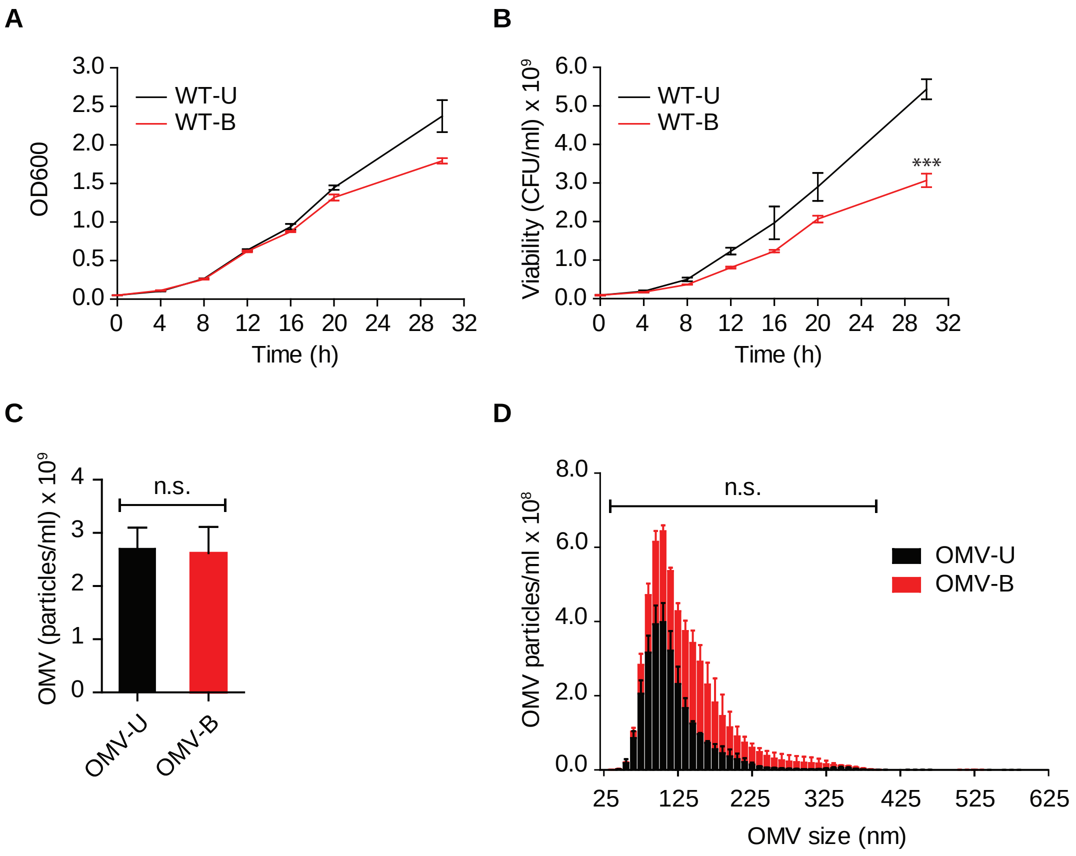

Figure S1. A-B. Growth curve of *C. jejuni* in response to ox-bile. *C. jejuni* was grown in MHB containing 0% (WT-U) or 0.025% ox-bile (WT-B) to reveal differences in growth over time up to 30 h. Bacterial growth was measured by OD600 (**A**) and bacterial survival was determined by viable count (**B**) at indicated time points. Data presented as the mean ± SEM for three independent experiments. ***$p \leq 0.001$; (one-way ANOVA followed by Bonferroni post-test). **C-D.** Size distributions of OMVs measured by nanoparticle tracking analysis of OMVs isolated from untreated (OMV-U) and 0.025% bile-treated (OMV-B) *C. jejuni*. (**C**) Total concentration of OMV presented as particles/ml. (**D**) Concentration of OMV for individual size presented as OMV particles/ml. Data presented as the mean ± SEM for three independent experiments. n.s.: not significant; (Mann-Whitney test).

Figure S2

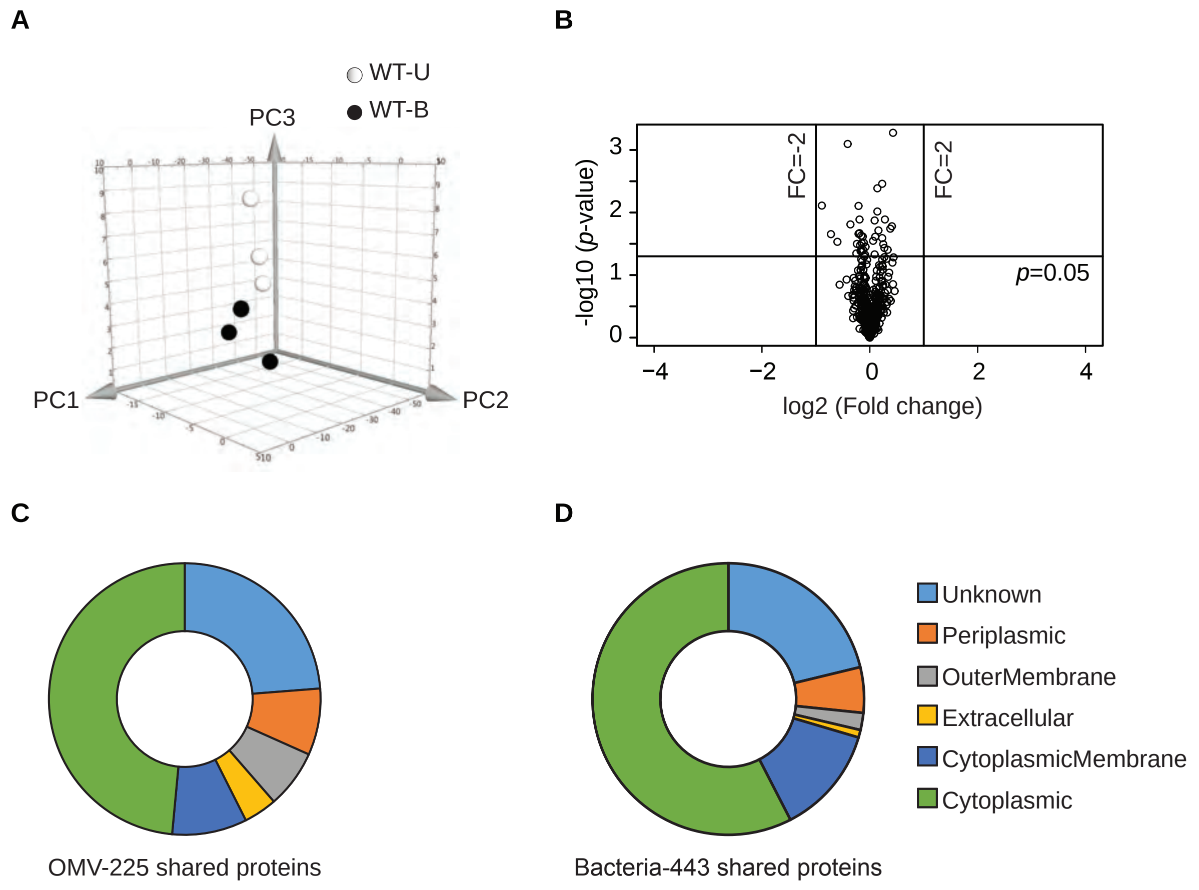

Figure S2. A. Principal component analysis showing the relative protein abundance profile of *C. jejuni* bacterial samples grown at 0% (WT-U) or 0.025% ox-bile (WT-B) for 20 h in a 3D graph with PC1, PC2 and PC3. The data were mean centered and log transformed. The three biological samples of each type were clustered based on the variance and correlation among them. **B.** Volcano plot of the complete set of shared proteins detected by proteomic analysis of bacterial samples. Each point represents the difference in expression (fold-change) between WT-U and WT-B plotted against the level of statistical significance (*p*-value). Solid lines represent differential expression. **C.** Subcellular localization of *C. jejuni* OMV proteins shared between OMV-U isolated from untreated and OMV-B isolated from bile-treated *C. jejuni*. **D.** Subcellular localization of *C. jejuni* bacterial proteins shared between WT-U and WT-B.

Figure S3

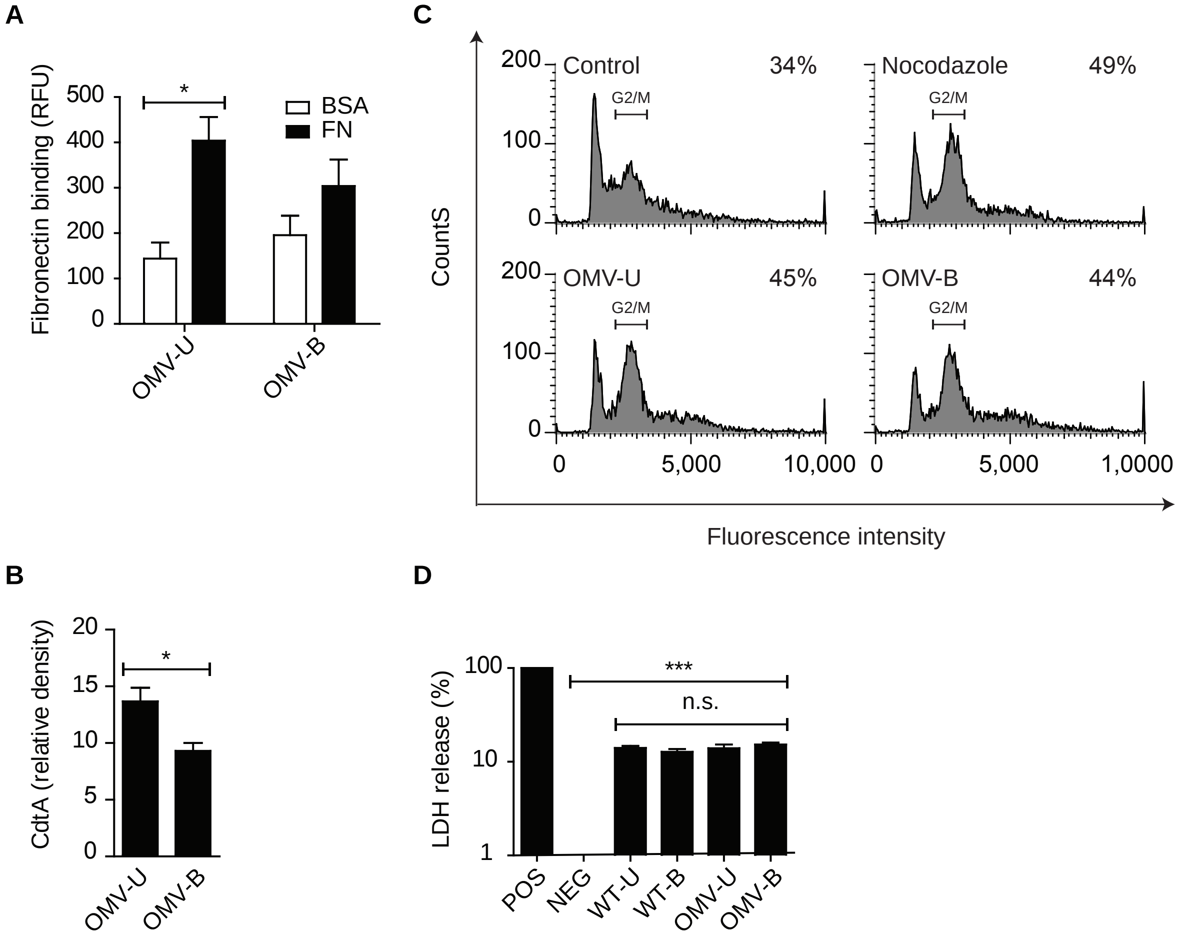

Figure S3. A. Binding of *C. jejuni* OMVs to fibronectin. OMVs released from untreated (OMV-U) or bile-treated (OMV-B) *C. jejuni* were examined by ELISA for the ability to bind to fibronectin (FN). Data presented as the mean ± SEM for four independent experiments. *$p \leq 0.05$; (one-way ANOVA followed by Bonferroni post-test). BSA was used as a negative control for binding. **B.** Western blot analysis of Cdt-A in OMV-U and OMV-B. The columns show densitometric determination of Cdt-A presented as relative density, mean ± SEM of three independent experiments. *$p \leq 0.05$; (Student's *t*-test). Uncropped full length blot for all three experiments used for densitometric quantification is shown in the Supplementary data Fig. S4B. **C.** G2/M phase arrest in HCT-8 epithelial cells. Cells were left untreated or stimulated with 10 µg/ml OMV-U or OMV-B for 24 hours at 37°C. G2/M phase arrest was analyzed by flow cytometer. Nocodazole (1µM) was used as a positive control. **D.** LDH release by LS174T epithelial cells in response to bile-treated *C. jejuni* and OMVs. Cells were left uninfected or infected with either *C. jejuni* 81-176 at a MOI of 100 grown in the presence (WT-B) or absence (WT-U) of 0.025% ox-bile or 10 µ g/ml OMV-U or OMV-B for 24 hours at 37°C. The cytotoxic effect on the LS174T cells was measured by quantifying the release of cytosolic lactate dehydrogenase (LDH) as a measure of cell damage. Uninfected cells represented 0% cytotoxicity (NEG), and total lysis of cells following treatment with 1% Triton X-100 represented 100% cytotoxicity (POS). Difference in cytotoxicity compared to WT-U. Data presented as the mean ± SEM for three independent experiments. ***$p \leq 0.001$, n.s.: not significant; (one-way ANOVA followed by Bonferroni post-test).

Figure S4

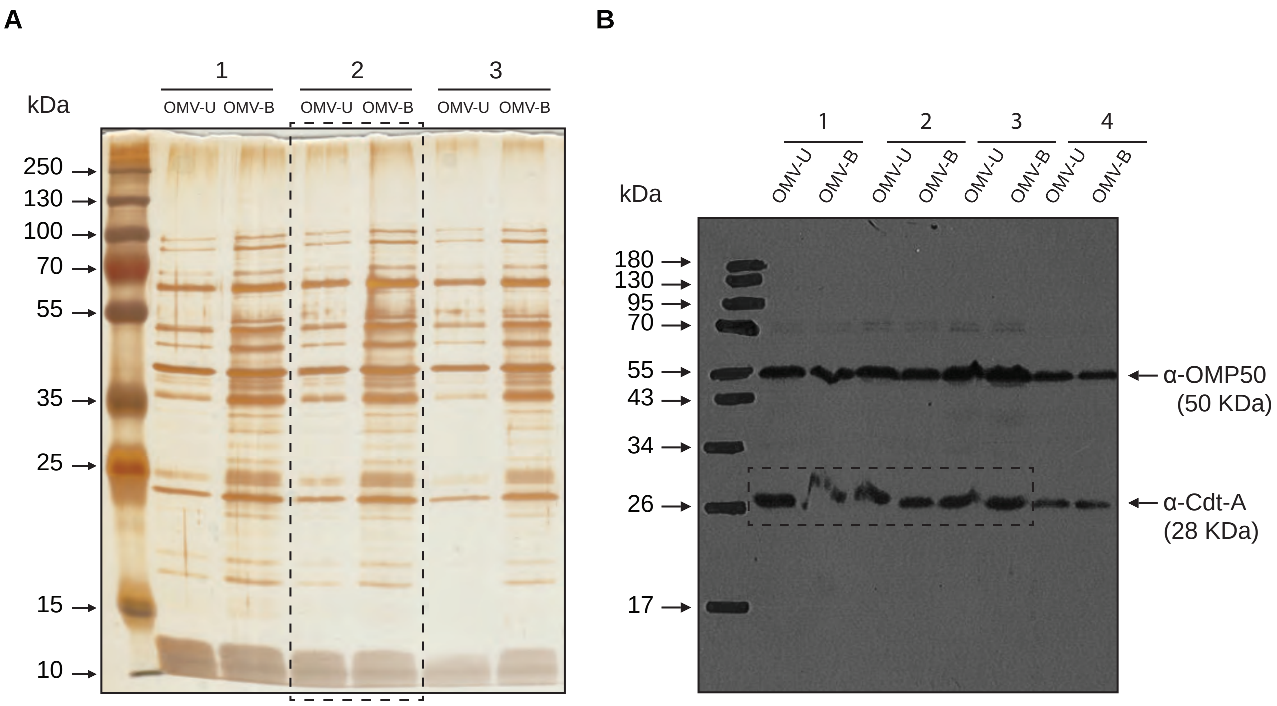

Figure S4. A. Original gel for Figure 2A. Silver stained gel of three independent experimental sets of OMV-U and OMV-B. Gel inside dotted square is shown in figure 2A. Protein size marker Page ruler plus no 26619 (Thermo). **B.** Full length image of Western blot for the three experiments (inside dotted square) used for densitometric quantification of Cdt-A in figure S3B. Protein size marker Page ruler plus no 26616 (Thermo). OMP50 was used as loading control.
